# Air Medical Evacuation of Nepalese Citizen During Epidemic of COVID-19 from Wuhan to Nepal

**DOI:** 10.31729/jnma.4857

**Published:** 2020-02-29

**Authors:** Bibek Rajbhandari, Naveen Phuyal, Bikal Shrestha, Moon Thapa

**Affiliations:** 1 Nepal Police Hospital, Maharajgunj, Kathmandu, Nepal; 2 Nepalese Army Institute of Health Sciences, Kathmandu, Nepal

**Keywords:** *Novel Corona virus*, *evacuation*, *Wuhan*, *Nepal*

## Abstract

In December 2019, the world was disrupted by the news of a new strain of virus known as Novel Corona virus, taking lives of many in China. Wuhan, the capital of Central China’s Hubei province is said to be the place where the outbreak started. The city went on lockdown as the disease spread rapidly. After the lockdown, most countries like India and Bangladesh airlifted their citizens who were studying in Wuhan. Similarly, Nepal also has many youth studying medicine in Wuhan. Pleas for help from the students reached the government. This was the first encounter of such experience for Nepal government. With the help of Ministry of Health, Ministry of foreign affair, Health Emergency Organizing committee, Epidemiology and Disease Control Division, Nepal Army Hospital, Nepal Police Hospital, Waste Management team, Nepal Ambulance service, Tribhuwan international Airport Authorities and Royal Airlines the government of Nepal planned, organized and successfully brought back all the 175 students on 15 th February 2019 from Wuhan, China. The present article aims to share the experience, the challenges faced and recommendations for future similar cases.

## INTRODUCTION

An outbreak of corona virus in Wuhan, China devastated the whole world in December 2019. Ban of travel in and out of China was discouraged and terror of the disease spread through countries. As Wuhan went down on a lockdown and panic-stricken citizens stayed in their houses waiting for the nightmare to be over, Nepal had another major issue to tackle.

Since past two decades’ students from Nepal have been going to China for further studies especially Bachelor in Medicine and Surgery(MBBS). The five-year course with various scholarships and other states of art technology -based education has been a lucrative offer for the Nepalese youth. Nepalese students fock in hundreds every year for a degree to various parts of China but this year they had no idea of what was in store for them. As the disease spread rapidly, countries including Sri Lanka, India, Malaysia airlifted their students from danger. But a low-income country like Nepal was under pressure and scrutinized for not being able to do the same.

Pleas for help through phone calls, social media and emails started to arrive as the students were under lockdown in their dorms with limited food and water supply. Worried parents back in Nepal started asking the government to bring back their children home, to safety. As the number of death tolls kept rising and the fear of the new strain of virus spread Nepalese students waited helplessly.

Back in Nepal, an urgent meeting was held in Singha Durbar, Kathmandu. A team of experts from Nepal Army Hospital, Nepal Police Hospital, Epidemiology and Disease Control Division, Health Emergency Organizing Committee met with the Prime Minister’s officials. Within the next couple of hours, a work plan was created to bring the students home. It wouldn’t be a simple pickup drop off plan. It had to be meticulously planned to look at all details and Nepal had never experienced this kind of situation and this would be completely new challenge for the concerned authorities.

Over the next few days, a lot of planning was discussed until a final plan was made. Next work division was done with team leaders for each phase. There would be four phases: Evacuation, Screening, Isolation and Quarantine.

For the first step, evacuation guidelines from the CDC and WHO were revised. With limited resources, manpower and technology it was difficult to maintain the universal guideline. Based on many literature reviews and consultations with experts a basic ground rule was made which was minimum contamination. The second was consultation with the airline crew and the aeronautical engineers as aerodynamics and inflight instructions played a major role in the 4-hour flight from Wuhan to China. It was decided to have minimum technical and medical staffs to maintain minimum touch technique to avoid contamination in the plane.

## EVACUATION PLANNING: PLANNING (PHASE I)

An urgent meeting was held in the PM office. The objective was the early evacuation of the students studying in Wuhan, China. The students had to be brought home safely then isolated and quarantined. This was a completely new task, there were no department or a body of government who handled these kinds of situations. It was stressful as time was running out and we didn’t know the magnitude of the disease very well. Many questions arose, what if the students were already infected, what if they were carriers of the disease, what if we bring the disease home, what if the medical or aeroplane crew catches the virus, will doctors that we assign be ready to get on that plane, who will risk their lives and more importantly are we putting everyone at risk. After major discussions and consultations, a decision was made, a list of names was put on the whiteboard. 4 teams were instantly created. Surprisingly energy flowed in, and more than expected volunteers from Nepal Army and Nepal Police offered their service.

## WORK DISTRIBUTION (PHASE II)

After the division of medical teams, work distribution was done. There would be evacuation, isolation, screening and quarantine. The responsibilities were each then assigned. Nepal Police DySP Dr.Bibek was in charge of the evacuation of students from Wuhan, Dr.Bikal Shrestha from Nepal Army was in charge of the screening process, and Dr.Naveen Phuyal from Nepal Army was in incharge of isolation and quarantine. Each team leader got their team ready and a short training was conducted for each phase.

## WASTE MANAGEMENT TEAM (PHASE III)

Another vital stakeholder in this operation was the waste management team. The waste produced in the plane, during the flight and later during the stay in quarantine had to be managed and disposed of properly. We lacked proper equipment and faced major setbacks. The plastic water bottles couldn’t be placed in autoclave machine with the caps on, each individual had to take the cap off before disposal, plastic bags where each individual disposed their food boxes couldn’t also be placed in the autoclave machine hence after much discussion a brown paper bag was decided instead of a plastic bag. There were so many things to consider, so many information to be gathered, and everything had to be meticulously planned, not missing any minor detail.

## LOGISTIC TEAM (PROCUREMENT AGENCIES)

In a low-income country like Nepal, the team did not expect to have everything in need and expected to very much adjust. But to everyone’s surprise, the logistic team was very supportive and provided almost everything that was needed. A budget was set and everything needed and that was available was provided. During this time, disposable masks were not easily available, and yet the teams were provided masks that were collected over days. The personal protection equipment provided was up to date. Waste disposal machines were brought immediately when it was realized the ones available couldn’t handle the tons of waste that were going to be created. 5 new mobile phones were bought and distributed among team leaders with sim cards and internet packages for the collection of photos of self-declaration forms (SDF). As the SDFs would be disposed of the photos would be sent to dedicated g-mail and Whatsapp for decoding and saved in case for contact tracing of the passengers. The mobile phones were also then placed in autoclaved and disposed of. 5 infra-red non touch thermometers were also provided by the logistic team on a short notice for the monitoring of temperature of the passengers. The 5 infra-red thermometers were also disposed of properly after its work was complete.

Training team (EDCD) for all medical as well as other team regarding PPE, personal. A class was held for the airline crew team at TIA. Everything from aerodynamics to medical procedure was discussed.

Shortly Nepal Ambulance team was also recruited and trained for the emergency. Ambulance system was made available for 24 hours. Transport Team formed of bus drivers only to maintain minimum contact. Disinfection process of airplane, bus and ambulance was planned by chemical Kala 1.4. The transport vehicles were also in quarantine and not in use as per protocol.

National laboratory capacity was strengthened to prepare for huge population arriving from Wuhan, and in case of an epidemic. As soon as the quarantine protocol was finalized, the search for an isolated place that could house all 175 students as the quarantine area started. After a thorough search and lots of meetings Kharipatti, Bhaktapur was finalized as the quarantine area. It was the perfect location as it was easily accessible but still not inhabited densely, the buildings were almost empty which helped as minimum furniture was planned to avoid contamination, the building already had running water and electricity, the enclosed area was spacious which would help the students to feel comfortable and not in a confinement. Once the location was fixed the planning for the stay started as in dedicated Isolation beds were allocated as the first step.

Medical Information Update team was added to the whole team to update about the status of the spread of COVID-19 and its new change and information. Risk communication was being planned simultaneously. Inter-sectorial authorise the connection and dedicated contact numbers were there, communication was strong between each team.

Possible challenges of weather variation, in our case rain, instead of one plane there could be two planes to adjust the students, if any students developed symptoms inflight and just everything possible that might occur a Plan B was prepared and discussed.

The draft formulation was revised and presented to the authorities.

### Resource mobilization:

There were two phases in resource mobilization:

1. Planning

2. Implementation

Financial resource planning: Fund was provided by the government, hence there was no hassles to mobilize the funds. Situation analysis budget outcome in case if not sufficient while implementing the plan, a contingency plan was formed for the specific.

Human resources: Every team consisted of Nepal Army and Nepal Police personnel. Since these were bodies of discipline and following chain of command it was easier to mobilize. Prompt action could be taken and punctuality was highly maintained. The job description of the team was well defined.

Local resource utilization: In case of shortage of any equipment from personal protective equipment, disinfectants local producers were allocated and were also sought to manufacture at a large level. For isolation, Patan Hospital, Sukraraj Tropical and Infectious disease Hospital and other satellite hospital were considered. These hospitals were prior informed and alerted.

Formulation of guideline principles for action plan development were formed and presented. Continuous monitoring and evaluation of the action plan in an interval of 3days, any changes seen in evaluation, the strategy was modified.

Lesson learnt: All planning, implemented steps, procedures were documented safely for future endeavours. In case of similar situations in future, these documented plans and implementations could provide information and guide in future challenges.

## PREPAREDNESS

The greatest advantage the team had, was proactiveness. It felt surreal to see each and everyone in the team was so full of energy and thinking proactively. It was an indescribable feeling that everyone felt during work. It is not sure if this is patriotism or passion for work or just anything but what the team felt was also witnessed by every single person who came to work at the site. Intersectional coordination was performed smoothly with dedicated contact details 24 hours.

For every strategy/situation/situation and its possible failure, a plan B was formed for backup.

Another vital role was played by the media. In order to avoid fake news, misconception about COVID-19 and its transmission, about the work being planned or anything related, a clear and strong communication with the media was very necessary. While communicating with the media it was very necessary to explain the ongoing situation in order to prevent unwanted fear escalating among the general public. Many controversary had arisen amidst the planning regarding the quarantine site and it needed to be cleared with information and not forcefully. We were working for the wellbeing of the public and in no way trying to harm them, and this message had to reach the mass loud and clear.

Possible loopholes were early discussed and managed. Role of each allocated team and personnel were defined and responsibilities timely checked. Review of regulatory requirements were done time and again. Mock drills were performed with airline crew, medical team and the transport team. The entry point, the exit point, the use of back door in case of symptomatic passenger, the minimum contamination practice and many other were included.

It may seem like very minor details and one would assume that it would be easily followed but on the ground, in reality, we were very far from it. One single mistake and this could lead to unimaginable chaos. The stakes were very high but the preparedness was even stronger. This team left no stone unturned and took into consideration every minute detail and information. This was a preparation Nepal had never witnessed before with the best of the country’s best involved.

## PROCEDURES:

**Evacuation step was divided into further 5 steps:**

1. Tribhuwan International Airport (TIA) management before the flight (At Kathmandu)
2. On the way to Wuhan flight management (Kathmandu to Wuhan)
3. Wuhan Airport management (At Wuhan)
4. Wuhan to Kathmandu flight management (Wuhan to Kathmandu)
5. After landing in TIA management. (At Kathmandu)

**Kathmandu to Wuhan**

1. Tribhuwan International Airport management before flight:

- Fill up of Self Declaration Form^1^ (SDF) by Air Medical Team (AMT)
- Temperature measurement of all team members
- Debriefng of the medical evacuation by AMT commander
- Placement of food, drinking water, mask, sanitizer, rubbish bag, clear bag, pen, SDF, necessary information leafet on their respected seat
- Re-examine the checklist meticulously

**2. During Flight:**

- Inspection of the aircraft diligently by the commander
- Storage area, AMT and passenger seats and toilet allocation

**Wuhan**

3. At Wuhan Airport:

- Communication with the ground staff for Personal Protection Equipment (PPE) preparation before boarding of passenger
- Emptying bowel and bladder and use of adult diaper before PPE
- Passengers were asked to board the plane 20 persons at a time, maintaining one-meter distance while boarding.^2^

**Cabin crew announcements:**

- To be seated in the exact seat allocation.
- All required materials are on their seats, to minimize movements around the plane.
- Use mask for whole flight duration.
- Announcements to check the leaflets (for information regarding hand rubbing technique to use sanitizer and handwashing technique) were made frequently (30 minutes’ interval).
- Toilets on the plane were allocated for passengers with symptoms and without symptoms. (symptomatic/ asymptomatic/ crew)
- All recommendations as per medical protocol^3,4,5^ to decrease possible contamination to be followed.
- Ask traveler to fill up Self Declaration Form.
- Screening after getting off from the plane, quarantine information, separate mode of travel plans for symptomatic and asymptomatic.
- Passengers were asked to switch off mobile phones and “not in airplane mode” along with other devices beside medical equipment to minimize contamination (leaflets).
- Clean bowel and bladder before departure (in leaflets)
- Bring rubbish bag containing food remains or cutlery, water bottles with cap open and old mask when they get off from plane (in leaflets)
- Bring leaflets, SDF form, boarding pass inside clear file during screening in departure (in leaflets)
- Luggage managed and dropped at quarantine center.
- Repeated messages regarding hand rubbing and washing with minimal walking.

**Wuhan to Kathmandu During Flight (Return):**

1. Application of duty roster (1-hour duty rotation of 1 crew member and 1 medical staff) with documentations.
2. Monitoring of passengers for 30 minutes by each crew and medical staff (if suspicious, move them to back of the plane, tag symptomatic seat)
3. Maintain register by the commander.
4. Cabin crew and medical staffs will be under the control of Medical Commander for medical purposes as per command system.

**Seat allocations and its rationale:**

“Fixed Seat Allocation”^6^ of passenger ensured by AMT. If possible, symptomatic should be at least 1 meter

1. Seating started from 2 rows (2 meters) away from the exact position of the cabin crew stands during announcements to avoid contamination.
2. The medical team were seated in the business class area and their seats were also fixed.
3. If any passengers developed any kind of flu symptoms they were moved to the back of the aircraft where there is negative pressure which flows the air to the back of the plane. Separation of the asymptomatic and the probable symptomatic passenger were done by 2 rows of seats again.^7^
4. At the back of the plane the symptomatic will be seated next to the window as the location of air suction is at this point, in contrast to the passengers seated in the middle row whose air will be circulated in the plane causing more contamination.
5. There were 8 HEPA filters in the plane, which were checked before takeoff and found up to date. The HEPA filter, filters the air of the plane and circulate clean and filtered air back to the plane.
6. Contact tracing was the motive behind the seat allocation of each passenger in case of future positive cases. Coding was done of the Self declaration forms as to maintain confidentiality; decoding was done on the basis of boarding pass.

**Figure 1:**
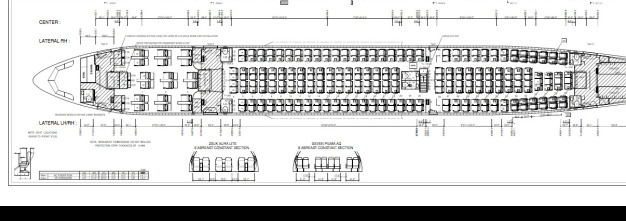
Anatomy of airbus.

**Figure 2:**
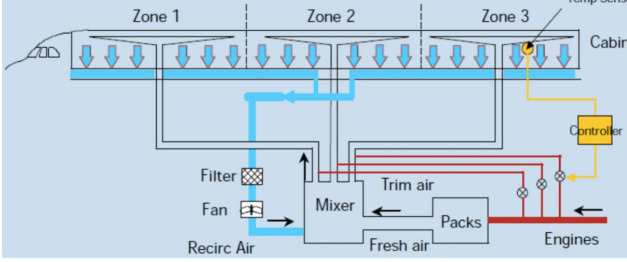
Recirculation airflow system of airbus.^7^

**Figure 3:**
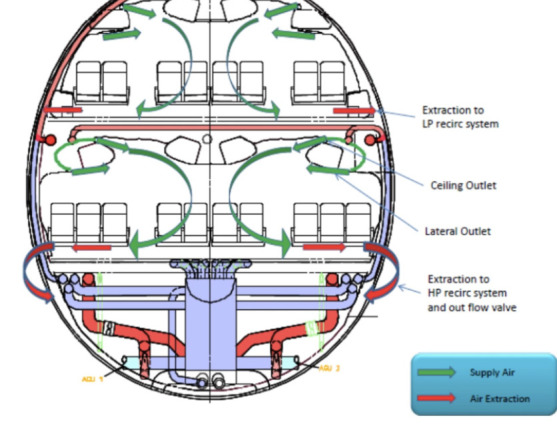
Ventilation system of airbus.^7^

**Figure 4:**
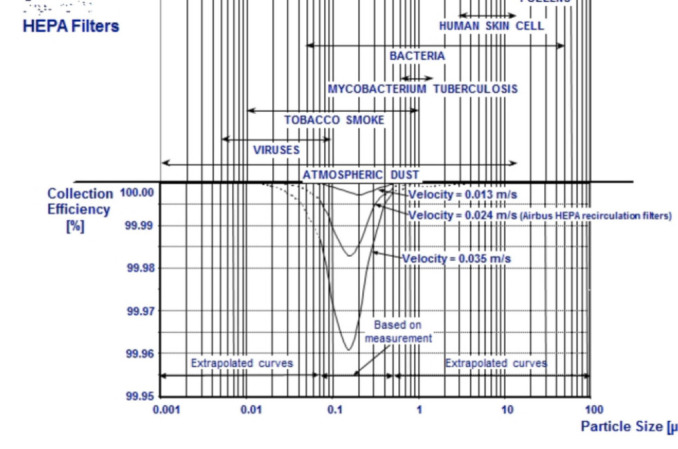
HEPA filter.^7^

## AFTER LANDING:

1. If anyone is sick, should give information to ground staff and exit through back/ symptomatic door earlier than the rest of the passengers and AMT staffs on board.
2. If no one shows any symptoms during the flight, the front door can be opened. AMT staffs will get off the plane prior to the passengers as per protocol.
3. During disembarkation, ask passengers to come serially 4-5 people at a time maintaining 1-meter distance. Send passenger to the screening/doffing bay for screening with the “Self-Declaration form” and waste products.
4. Hand over all documents, biohazard bags to screening team leader.
5. After all the passengers are disembarked, report to screening station and disinfectant authority by commander. Remove PPE, dispose appropriately, perform hand hygiene by AMT member handover to screening team.
6. AMT member departure as per protocol (doffing and screening)

**Table 1:** Chronological Order of Events

Date	Place / Country	Remarks
31 December 2019	Wuhan, China	A pneumonia of unknown cause reported to WHO country office
7^th^ January		Novel corona, temporary name given
11^th^ January	China	First death due to novel corona
13^th^ January	Thailand, Nepal	1^st^ case identification in Nepal 1^st^ imported case, Cross border spread
20^th^ January	China	Human to human transmission
30 January		Outbreak was declared a public health emergency of international concern
1^st^ February 2020		1^st^ nCoV-19 Air evacuation from Wuhan to India
1^st^ February 2020	Emergency meeting at PM’s residence, Nepal	Emergency meeting at PM’s residence ( planning for evacuating Nepali students from Hubei province) Searching probable place for quarantine
2^nd^ February 2020		Searching probable place for quarantine
2^nd^ February 2020	Meeting at Chief secretary’s office, Singh durbar	Regarding evacuation process of Nepali students from Hubei Province, China; keeping them in Quarantine; detail planning
9^th^ February 2020	Meeting with airport authorities and visit to airport	To understand aerodynamics and medical adjustment for air medical travel protocol
9^th^ February 2020		Evacuation Team identified
10^th^ February 2020	EDCD, Nepal	Training of the team
11^th^ February 2020	Meeting at HEOC, Nepal	Hospital preparedness and medical response readiness for 2019-nCoV
13^th^ February 2020		Medical teams, hygiene team, other personnel oriented and trained. Donning and doffing of PPE practiced.
14^th^ February 2020	TIA, Nepal	Rehearsal for evacuation
14^th^ February 2020	Kharipati, Nepal	Rehearsal for Quarantine
15^TH^ February 2020	Nepal, China	Evacuation team left KTM
16^th^ February 2020	Nepal	175 Nepalese citizens evacuated from Wuhan received at TIA
16^th^ February 2020	Kharipati, Nepal	Evacuated citizens received at Quarantine centre, Kharipati

**Figure 5:**
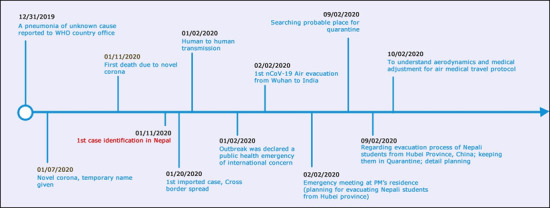
Planning Timeline of Air Medical Evacuation

## LESSON LEARNED AND WAY FORWARD:

**Lack of resources:** Nepal is a low-income country where everything is limited from money to manpower. During situations as these, medical team works in scarcity and use whatever is available. There were challenges to get the latest non touch temperature measurement technique to limit contact with the passengers, we do not have latest technology to dispose biohazard waste, and the updated evidence-based information regarding similar situations.

**Communication:** It would have been more efficient and effective if communication between management team and the people working on the ground was planned beforehand. In future a committee should be formed to handle such outbreaks where there are experts from Health Emergency Operating Centre, Home Ministry, Defence Ministry, Epidemiology and Disease Control Division team, waste management team, logistic authority team, airline crew including aeronautical engineers for information on airflow system, aircraft inbuilt system (each aircraft has a unique system that can be updated only from the manufacturer, so in order to add an audio-visual regarding the instructions to dispose water bottles and food packages, fill self-declaration form, wear the provided mask, etc. at least 10 days is required to update the aircraft system. If informed and planned accordingly this would have been possible and medical staffs and airplane crew’s contact with passengers would be minimized.

**An app of self-declaration form:** Instead of hard copy self-declaration, if we could have developed an app for the self-declaration forms, less contact would have been possible and there would be less waste to dispose.

**Maintaining the standards:** As it was a first encounter of such event for state, there was no standard guidelines and everything was planned based on expertise and literature review. Disinfecting the plane before and after flight, technical details that who should wear PPE, autoclave of the cutlery, disposal of the waste of the plane, manage the luggage of the passengers and how to disinfect them, technicality of quarantine procedures for passenger crew members including the cockpit member and the air medical teams, knowing the exact capacity of hub and satellite hospital for isolation, training of the PPE and waste management to AMT, screening team, ground handlers, ambulance and bus drivers and others, Proper and defined job description of the individuals.

## Conflicts of Interest:

None.
